# HSET overexpression fuels tumor progression via centrosome clustering-independent mechanisms in breast cancer patients

**DOI:** 10.18632/oncotarget.3475

**Published:** 2015-02-28

**Authors:** Vaishali Pannu, Padmashree C.G. Rida, Angela Ogden, Ravi Chakra Turaga, Shashikiran Donthamsetty, Nathan J. Bowen, Katie Rudd, Meenakshi V. Gupta, Michelle D. Reid, Guilherme Cantuaria, Claire E. Walczak, Ritu Aneja

**Affiliations:** ^1^ Department of Biology, Georgia State University, Atlanta, GA, USA; ^2^ Center for Cancer Research and Therapeutic Development, Clark Atlanta University, Atlanta, GA, USA; ^3^ Department of Human Genetics, Emory University School of Medicine, Atlanta, GA, USA; ^4^ Clinical Pathology & Anatomic Pathology, West Georgia Hospitals, LaGrange, GA, USA; ^5^ Department of Pathology, Emory University School of Medicine, Atlanta, GA, USA; ^6^ Department of Gynecologic Oncology, Northside Hospital Cancer Institute, Atlanta, GA, USA; ^7^ Department of Medical Sciences, Indiana University, Bloomington, IN, USA

**Keywords:** HSET, centrosome clustering, microtubule motor, cell-cycle kinetics, tumor progression

## Abstract

Human breast tumors harbor supernumerary centrosomes in almost 80% of tumor cells. Although amplified centrosomes compromise cell viability via multipolar spindles resulting in death-inducing aneuploidy, cancer cells tend to cluster extra centrosomes during mitosis. As a result cancer cells display bipolar spindle phenotypes to maintain a tolerable level of aneuploidy, an edge to their survival. HSET/KifC1, a kinesin-like minus-end directed microtubule motor has recently found fame as a crucial centrosome clustering molecule. Here we show that HSET promotes tumor progression via mechanisms independent of centrosome clustering. We found that HSET is overexpressed in breast carcinomas wherein nuclear HSET accumulation correlated with histological grade and predicted poor progression-free and overall survival. In addition, deregulated HSET protein expression was associated with gene amplification and/or translocation. Our data provide compelling evidence that HSET overexpression is pro-proliferative, promotes clonogenic-survival and enhances cell-cycle kinetics through G2 and M-phases. Importantly, HSET co-immunoprecipitates with survivin, and its overexpression protects survivin from proteasome-mediated degradation, resulting in its increased steady-state levels. We provide the first evidence of centrosome clustering-independent activities of HSET that fuel tumor progression and firmly establish that HSET can serve both as a potential prognostic biomarker and as a valuable cancer-selective therapeutic target.

## INTRODUCTION

About 80% of invasive breast cancers exhibit supernumerary centrosomes, a feature commonly referred to as centrosome amplification [1]. Most breast cancers harbor abnormalities in centrosome structure, function, or localization within the cell. These abnormalities can potentially cause cytoarchitectural distortion in cancer tissues with loss of cellular differentiation (anaplasia) via altered centrosome function in microtubule nucleation and organization [2]. Centrosome amplification can initiate tumorigenesis in *Drosophila* neuroblasts [3]; thus, it is becoming recognized that centrosome amplification is one of the primary causes of breast cancer and is not just a consequence of malignant transformation.

The presence of more than two centrosomes within a cell can pose a grave conundrum as it may lead to the assembly of a multipolar mitotic spindle, and the production of nonviable progeny cells due to lethal levels of chromosomal loss or gain (i.e., death-inducing, high-grade aneuploidy) [4]. However, cancer cells harboring extra centrosomes circumvent these catastrophic consequences and survive. The secret to their survival and success, as it turns out, lies in a clever tactic that cancer cells use to sidestep spindle multipolarity, viz., centrosome clustering, whereby the excess centrosomes are artfully corralled into two polar foci to enable formation of a pseudo-bipolar mitotic spindle [5, 6]. During a preceding, transient, multipolar state, merotelic kinetochore-microtubule attachments occur, thus engendering low-grade whole chromosome missegregation that could be ‘tumor-promoting’ [7].

HSET/KifC1, a minus end-directed motor protein that promotes microtubule cross-linking, sliding, bundling and spindle pole focusing, has been recently identified as an essential mediator of supernumerary centrosome clustering in cancer cells [8]. HSET has also been shown to be indispensable for the clustering of acentrosomal microtubule organizing centers (MTOCs) whose production tends to be hyperactivated in cancer cells. HSET knockdown in cells with supernumerary centrosomes causes excess centrosomes to be scattered by pole-separating forces, leading to rampant spindle multipolarity and cell death [9]. By contrast, HSET function appears to be non-essential in healthy somatic cells due to the presence of two centrosomes that shoulder the responsibility of bipolar spindle assembly. In cells devoid of centrosomes, such as oocytes, HSET function is indispensable for the assembly of a fusiform bipolar spindle [10].

Recently, attention has converged on HSET as a potential chemotherapeutic target due to its intriguing association with malignancy. RT-PCR studies have shown that HSET's expression level in lung cancer is associated with increased risk of metastatic dissemination to the brain [11]. Docetaxel resistance in breast cancer is also suggested to be partly mediated by HSET [12]. *In silico* studies reveal that HSET expression is also higher in triple negative breast cancers compared to non-triple negative ones [13]. The differential dependence of cancer cells on HSET for viability and association of HSET expression with metastases-raise the tantalizing possibility that HSET may play a more important role in tumor progression than previously appreciated. However, more direct evidence of HSET's role in clinical progression of breast cancer and mechanistic studies revealing the molecular circuitry involved therein are lacking.

In this study, we evaluated HSET expression in breast carcinomas and examined its association with clinical tumor progression. Intriguingly, we found that HSET overexpression at the time of diagnosis was significantly associated with worse prognosis and overall survival. Exploration of its mechanistic role in tumor progression unmasked plausible centrosome clustering--independent roles of HSET underlying enhanced tumor cell proliferation and survival, and disease progression. Our results substantiate the idea that HSET could be an invaluable, cancer-cell selective therapeutic target and may serve as a prognostic biomarker for breast cancer.

## RESULTS

### HSET is overexpressed in variety of human cancers

**Figure 1:**
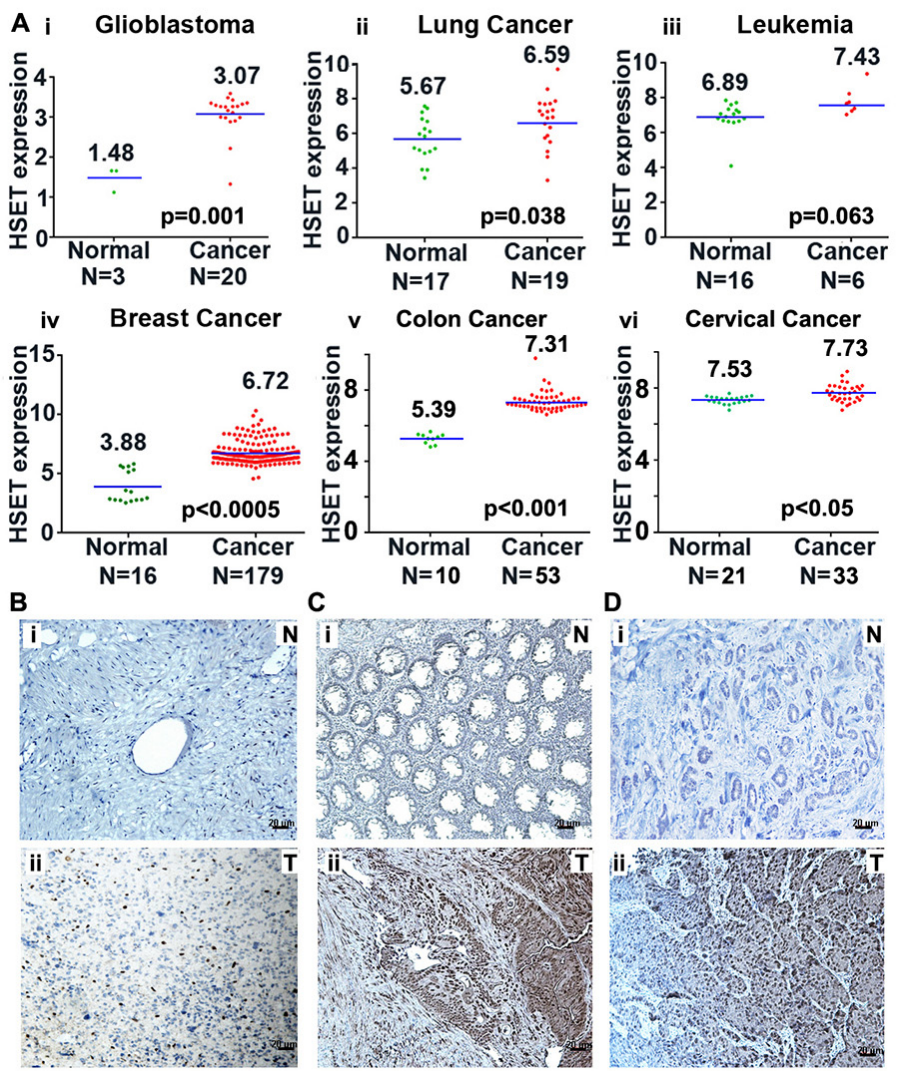
HSET overexpression in human carcinomas (A) Scatter plots depicting HSET gene expression in normal (green dots) versus tumor (red dots) tissues in (i) Glioblastoma, (ii) Lung carcinoma, (iii) Leukemia, (iv) breast carcinoma, (v) colon carcinoma and (vi) cervical carcinoma. Data was obtained from one channel microarray available at GEO database. Robust multiarray normalization was performed to obtain the differences depicted in the plots. (B, C and D) Immunohistographs showing HSET expression in glioblastoma tissue where a representative normal tissue (N) (Bi) is compared to tumor tissue (T) (Bii), in colon tumor (Cii) versus adjacent normal (Ci) tissue, and in cervical tumor (Dii) versus adjacent normal (Di) tissue.

Given the crucial requirement of centrosome clustering mechanisms for the viability of cancer cells with extra centrosomes, we first wanted to examine the abundance of the clustering protein HSET in various cancers that harbor extra centrosomes. We performed an *in silico* gene expression analysis using publically-available microarray data to determine the expression level of HSET in various cancer tissue types. One-channel microarray data for glioblastoma, leukemia, lung and breast cancer patients with their normal sample pairs were collected from Gene Expression Omnibus (GEO) database [14]. Each of these samples were then Robust Multiarray (RMA) normalized [15], and their logarithm to base 2-transformed HSET gene expression values were plotted to determine the difference as shown in Fig. 1Ai-vi. Next, we determined differences in HSET gene expression for cancer and normal sample groups using two-tailed test of hypothesis. Our statistical results indicated higher HSET gene expression in glioblastoma, lung, breast, colon and cervical tumor samples as compared to their corresponding normal tissues. All these tumor types have been shown in various studies with exhibit significant degrees of centrosome amplification [16-25]. The average HSET expression for glioblastoma (N=20) and colon cancer (N=53) patients was found to be ~3-fold higher than normal samples (N=3 and 10, respectively) (p<0.005), followed by breast cancer patients (N=179) with more than 5-fold higher expression in tumors than in normal samples (N=16) (p<0.001). Our *in silico* results were consistent with observations from a previous study wherein HSET mRNA expression was significantly elevated in a broad panel of primary tumor tissue compared to corresponding normal tissue [9]. Our *in silico* data corroborate immunohistochemical analysis suggesting a significantly higher HSET expression in glioblastoma, colon and cervical tumors (Fig. 1Bii, Cii, Dii) as compared with their respective adjacent normal tissue samples (Fig. 1Bi, Ci, Di). These data suggest HSET overexpression as a general feature of cancers exhibiting significant centrosome amplification.

### HSET is overexpressed in human breast cancers

**Figure 2:**
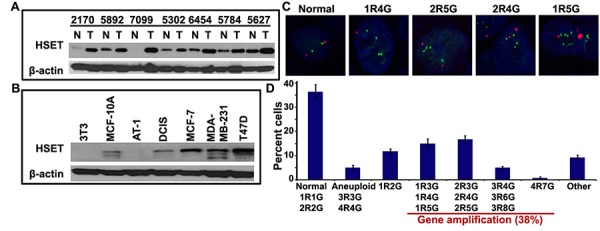
The HSET gene is overexpressed in breast cancers (A) Cell lysates from 16 paired clinical breast tumor tissues (T) and normal adjacent tissues (N) were analyzed for HSET protein expression by western blotting. Representative results of 7 paired samples are shown. (B) Immunoblot showing HSET expression in a) MCF10A series of cell lines representing a continuum from near-normal breast (MCF-10A) to pre-malignant (MCF10-AT1) to comedo ductal carcinoma *in situ* (MCF10-DCIS), aggressive breast cancer cell lines such as MDA-MD-231 and T47D and normal mouse fibroblast cell line, 3T3. (C) Representative confocal micrographs showing fluorescence *in situ* hybridization of two bacterial artificial chromosome (BAC) probes, one from the HSET locus on chromosome 6 (RPCI-11 602P21, green) and one from the chromosome 6 centromere (CH514-7B4, red), to paraffin-embedded primary breast tumor tissues. Amplifications of HSET were visualized as an increase in the number of green signals (denoted as G) relative to the number of red control centromere signals (denoted as R), where 1R1G and 2R2G represent normal HSET gene copy numbers and 1R4G, 2R4G, 2R5G, 1R5G,etc. represent instances wherein HSET gene locus is amplified. (D) Bar graph representation of various combinations of red and green copy numbers observed for HSET locus and chromosome 6 centromere as determined by visual quantitation from confocal images (p<0.05). 1R1G and 2R2G are considered normal copy numbers; elevated copy numbers but same ratio of R and G signals are considered as aneuploid (3R3G, 4R4G) and all other combinations with higher G-to-R ratios are considered to represent instances where the HSET gene is amplified.

Our *in silico* analyses of microarray data showed that breast cancers display significantly higher HSET expression (~5-fold) than corresponding normal tissue. In addition, given the pronounced occurrence of amplified centrosomes and centrosome clustering in aggressive breast cancer, we decided to focus our study on breast cancers. To explore the role of HSET in tumor progression, we examined whether HSET was overexpressed in human breast tumors. We immunoblotted 16 fresh-frozen human tumor samples and their paired adjacent normal tissues for HSET. An enhanced expression of HSET was observed in 10 tumor samples compared to their normal adjacent tissues and 7 representative normal/tumor sample pairs are shown in (Fig. 2A). The remaining 6 normal/tumor pairs showed negligible overexpression of HSET (data not shown). Additionally, HSET expression in most human breast cancer cell lines was much higher than in non-cancerous or pre-malignant cell lines such as NIH3T3 and MCF10 series (MCF10A, MCF10AT1, MCF10DCIS) (Fig. 2B), indicating that HSET overexpression typifies breast cancer cells.

Since higher HSET protein levels could arise either from an upregulation of transcription from the endogenous locus and/or an amplification of the locus encoding HSET, we decided to examine the copy numbers of the locus encoding the HSET gene in normal and breast tumor tissues. We performed fluorescence in situ hybridization (FISH) to directly evaluate HSET copy number per cell in paraffin-embedded breast tumor tissues. We hybridized two bacterial artificial chromosome (BAC) probes to primary breast tumor tissues, one from the HSET locus on chromosome 6 (RPCI-11 602P21, green) and one from the chromosome 6 centromere (CH514-7B4, red). Amplification of HSET was visualized as an increase in the number of HSET signals relative to the number of control centromere signals. We scored HSET amplification by FISH in four breast tumor tissues and found that, among them, three tumors exhibited HSET amplifications. No amplification of the HSET locus was observed in the normal adjacent tissues in these samples. We observed various types of copy number changes associated with HSET as shown in (Fig. 2C, D). FISH with the centromere probe indicated that most increases in HSET loci were not due to polyploidy of chromosome 6; rather, only 5% of cells were aneuploid. 38% of cells (500 cells each were counted from 2 tissue samples) showed 3 or more copies of HSET paired with only 1 or 2 copies of the centromere (Fig. 2D). More so, cancer cells isolated from fresh human breast tumor also showed HSET amplification (Suppl. Fig. 1). These findings indicate alterations in the HSET gene copy number during tumorigenesis. It is worth mentioning that HSET gene amplifications in specific breast tumor samples were correlated with increased expression of HSET protein in all those samples using immunoblotting methods (data not shown).

### HSET overexpression correlates with breast cancer progression and aggressiveness

**Figure 3:**
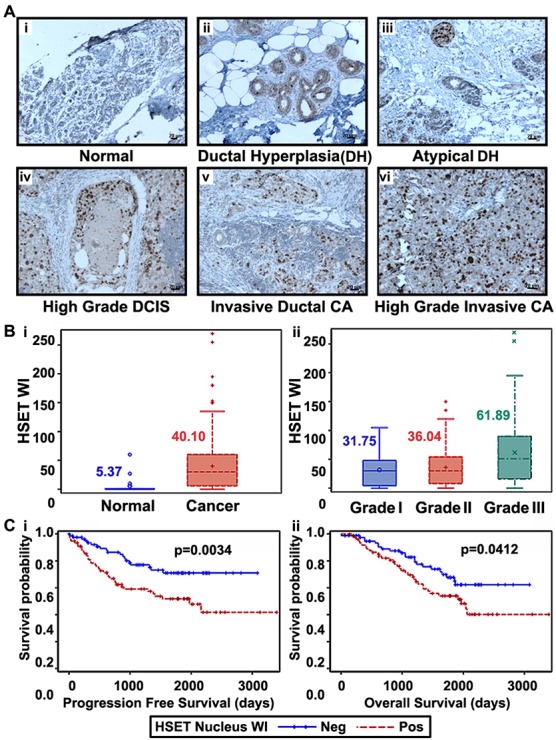
HSET overexpression correlates to increased aggressiveness and poorer clinical outcomes Immunohistographs showing HSET expression in (Ai) normal breast, (Aii) ductal hyperplasia, (Aiii) atypical ductal hyperplasia, (Aiv) ductal carcinoma in-situ, (Av) invasive ductal carcinoma, low-grade, (Avi) invasive ductal carcinoma, high-grade. Brown (DAB) color shows HSET staining. Intensities of nuclear HSET staining were quantified using image analysis Aperio Image Scope v.6.25 software. A weighted index score (WI) for HSET expression was calculated and was assessed in 339 breast cancer and 19 normal samples. Box-whisker plots showing (Bi) HSET WI score in normal breast and tumor samples, (Bii) HSET WI score across Grade I (n=40), Grade II (n=237) and Grade III (n=62) breast cancer samples. (Ci) Probability of progression free survival of 163 breast cancer patients with HSET nuclear expression above or below the median HSET weighted index (WI) value referred to as positive and negative, respectively (p=0.0034). (Cii) Probability of overall survival of 163 patients with positive and negative HSET weighted index (p=0.0412). Statistical analysis was conducted using SAS Version 9.3. Scale bar=10 μm.

Next we asked how HSET overexpression correlates with breast cancer progression and aggressiveness. Using an immunohistochemical approach, we stained a total of 60 clinical specimens with 10 cases each of normal breast, ductal hyperplasia (DH), atypical ductal hyperplasia (ADH), ductal carcinoma *in situ* (DCIS), invasive breast carcinoma (low-grade) and invasive breast carcinoma (high-grade). In consonance with our immunoblotting data, our immunohistochemical analysis showed that HSET is selectively overexpressed in human breast cancers with negligible or absence of expression in normal breast epithelia (Fig. 3A). Intriguingly, we observed a selective increase in nuclear staining of HSET in tumor samples. Among subtypes based on varying types and extent of intraductal proliferation, we found a progressive increase in HSET nuclear staining intensity and frequency from ductal hyperplasia (DH) (Fig. 3Aii) to atypical ductal hyperplasia (ADH) (Fig. 3Aiii) to ductal carcinoma *in situ* (DCIS) (Fig. 3Aiv). In invasive breast cancers (both low- and high- grade), HSET nuclear staining was remarkably intense, with a significant increase in the number of positively stained nuclei per field in high-grade cancers (Fig. 3Av,vi) compared to low-grade ones (Fig. 3Aii,iii,iv). A majority of normal breast tissue samples (85%) showed no staining for HSET, while the remainder showed very weak staining (Fig. 3Ai, data not shown). We then calculated a weighted index score (WI) for HSET expression as a product of the staining intensity score (+1, +2, +3) and percentage positive nuclei for each sample. HSET WI serves as an independent measure of the strength of HSET protein expression across all breast tumor specimens. Nuclear HSET WI values were then correlated with normal and tumor samples and also with the grade of tumor samples. Interestingly, nuclear HSET WI showed a strong correlation with increasing tumor grade in breast cancer (Fig. 3Bi,ii). Collectively, these observations indicate robust HSET overexpression in human breast tumors suggesting that abnormal HSET levels correlate with breast cancer development and HSET might play a role in progression of tumors into more malignant and aggressive forms. Having established a significant correlation between HSET expression and tumor differentiation, we next asked if there was an association of nuclear HSET WI with progression-free survival (PFS) and overall survival (OS) in breast cancer patients, using the biospecimens obtained from Emory Hospital with retrospective clinical information (n=163). While PFS was calculated as the number of days from diagnosis to the first local recurrence or metastasis if one occurred, or the last follow-up if the patient did not progress, OS was based on the number of days from diagnosis to death or last follow-up if death was not recorded. Nuclear HSET WI was also categorized into high and low groups based on the median. Irrespective of the receptor status (n=163), those with higher nuclear HSET WI (shown as HSET WI positive in Fig. 3Ci,ii) had significantly shorter PFS (p= 0.0034) and OS (p=0.0412) than patients with lower nuclear HSET WI (shown as HSET WI negative in Fig. 3Ci,ii), clearly demonstrating that higher nuclear HSET expression levels significantly correlate with poorer clinical outcomes. Multivariate analysis accounting for HSET nuclear expression, age, grade and receptor status (ER, PR, HER2) revealed that HSET nuclear expression and receptor status (negative) significantly correlated with both OS (p=0.030 for nuclear HSET WI, p=0.036 for PR negativity) and PFS (p=0.044 for nuclear HSET WI, p=0.003 for HER2 negativity). Mean HSET was significantly higher (7.82 vs 5.50, p <.0001) for triple-negative patients as opposed to non-triple-negative patients. In a wound-healing assay, we show that HeLa cells transiently overexpressing HSET show enhanced migration as compared to wild-type HeLa cells (Suppl. Fig. 2). These observations strongly suggest correlation of HSET nuclear expression with breast tumor aggressiveness.

### HSET overexpression is associated with enhanced cell proliferation

**Figure 4:**
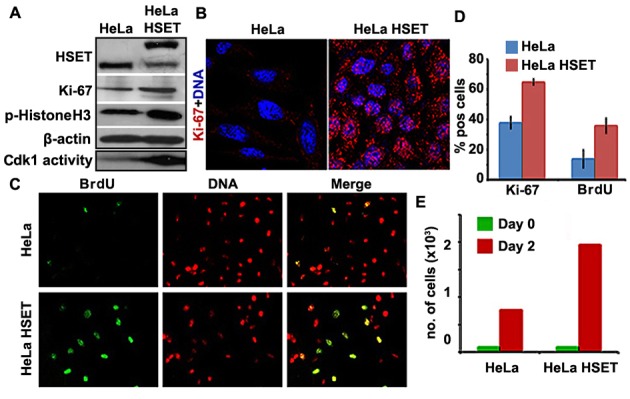
Cell proliferation is enhanced in HeLa cells that stably overexpress HSET (A) Immunoblots showing higher Ki67 and p-histone H3 in HeLa-HSET-GFP (denoted as HeLa HSET) cells as compared with HeLa cells. Kinase activity assay showed higher cdk1 activity in HeLa-HSET-GFP cells as represented by the immunoblot showing enhanced phosphorylation of Histone H3 by cdk1 as compared to HeLa cells. The two bands representing HSET expression correspond to the endogenous HSET levels (lower band) and the GFP-HSET levels (upper band). (B) Representative confocal immunomicrographs showing higher Ki67expression (red) in HeLa-HSET-GFP cells as compared with HeLa cells. (C) Randomly dividing HeLa-HSET-GFP and HeLa cells were incorporated with BrdU and immunostained with anti-BrdU antibody (green) to visualize the cells traversing S-phase. Representative immunofluorescence images showing higher BrdU incorporation in HeLa-HSET-GFP cells. (D) Bar graphs depicting the percent cells that are Ki67or BrdU positive in HeLa and HeLa-HSET cells. (E) Bar graphs representing number of cells in cell proliferation assay counted by trypan blue at Day 0 and Day 2 of seeding.

Since elevated HSET expression exhibits a strong correlation with clinical development and progression of cancer, we next asked if high HSET levels had any impact on the kinetics of cancer cell proliferation *in vitro*. To this end, we used HeLa cells stably transfected with HSET-GFP to examine and compare the levels of various cell proliferation markers in HeLa-HSET-GFP and HeLa cells. Using immunoblotting methods, we found that levels of Ki67 (found in G1, S, G2 and M-phases of the cell cycle but is absent in G0 cells) was substantially elevated in HeLa-HSET-GFP cells compared to wild-type HeLa cells (Fig. 4A). This finding was consistent with the strikingly higher Ki67 labeling index observed in HeLa-HSET-GFP cells via immunostaining (Fig. 4B). This observation is noteworthy since the Ki67 labeling index often correlates with the clinical course of cancer, owing to which the proportion of Ki67-positive cells in a cell population has strong prognostic value for survival and tumor recurrence in cancer patients [26, 27]. Immunofluorescent staining for BrdU, a marker for cells undergoing S-phase, also showed that a greater proportion of HeLa-HSET-GFP cells were BrdU-positive compared to HeLa cells (Fig. 4C). A visual quantitation of these observations revealed significantly elevated levels of Ki67 expression and BrdU incorporation in HeLa-HSET-GFP cells as compared with HeLa cells (Fig. 4D). We also observed enhanced cdk1 activity and higher expression of phosphorylated histone-H3 in HeLa-HSET-GFP cells compared to HeLa cells, which is indicative of a larger proportion of cells in the HeLa-HSET-GFP line undergoing M-phase (Fig. 4A). All these lines of evidence strongly advocate for a pro-proliferative role for HSET overexpression in the cellular context of cancer cells. HeLa-HSET-GFP cells also displayed significantly enhanced cell proliferation capacities when compared to HeLa cells in trypan blue assay. Equal numbers of each cell type were seeded on day 0 and were allowed to grow for 2 days (48h), and the number of cells were counted using trypan blue. Based on the data, the doubling time of HeLa-HSET-GFP cells was found to be ~11h as compared to ~16h for HeLa cells (Fig. 4E). We also performed colony formation assay with HeLa cells transiently transfected with control vector, HSET-GFP plasmid and HSET-GFP siRNA. HSET OE cells were able to form significantly higher number of colonies as compared to cells transfected with control vector. Fewer colonies were observed upon HSET knockdown (KD) (Suppl. Fig. 3). Similar proliferation effects were confirmed by colony formation assay in another breast cancer cell line, MDA-MB-231 upon transient HSET OE and KD (Suppl. Fig. 3). Taken together, these data demonstrate that cells overexpressing HSET display enhanced cell proliferation, and suggest that HSET overexpression may confer significant proliferative advantages to cancer cells.

### HSET overexpression leads to accelerated cell cycle kinetics

**Figure 5:**
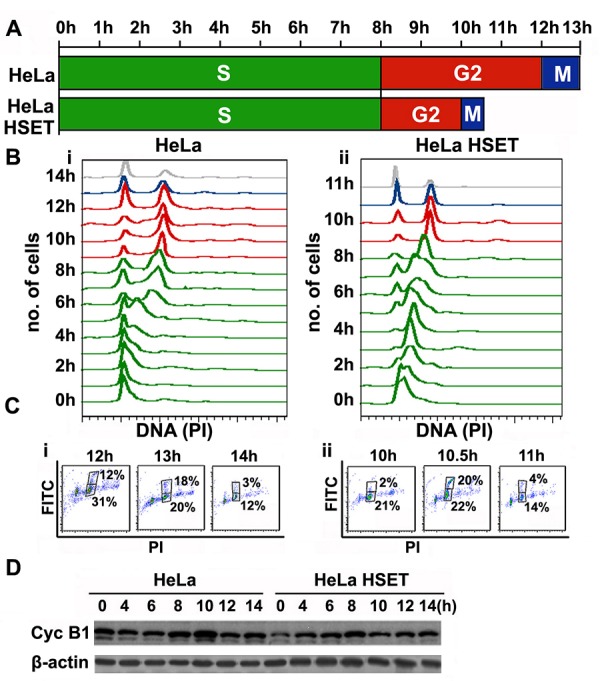
HSET overexpression accelerates cell cycle kinetics (A) Schematic depicting duration of each cell cycle phas in HeLa and HeLa-HSET-GFP cells assessed by flow cytometry following synchronization at G1/S border by single thymidine block.. (B) Cell cycle histograms representing cell cycle profiles of synchronized (i) HeLa and (ii) HeLa-HSET-GFP cells from the point of thymidine block release (0h) to the point after mitotic exit (14h and 11h, respectively). (C) Dot plots of PI (DNA) vs FITC (MPM-2) showing cells in G2- (lower box) and M-phase (upper box) specifically during the time of mitotic exit in (i) HeLa and (ii) HeLa-HSET-GFP cells. Two-color scatter plot (PI vs. GFP) shows two box gates, where the lower box represents G2 population (PI-4N and FITC negative) and upper box represents M population (PI-4N and FITC positive). G2/M population was represented by double the intensity of PI (4N) as compared with G1 population (2N). Mouse anti-MPM-2 antibody tagged with anti-mouse Alexa-488 secondary antibody was used as a mitosis-specific marker, to distinguish G2 and M populations. The time for mitotic exit was determined by assessing the population in the upper gate of the 2-color scatter plot. A sudden surge in the proportion of mitotic population followed by a rapid fall indicated the time of mitotic exit. 13h was observed as the time of mitotic exit for HeLa cells whereas, 10.5h was the time of mitotic exit for HeLa-HSET-GFP cells. (D) Immunoblots showing cyclin B1 protein levels in synchronized HeLa and HeLa-HSET-GFP cells following release from thymidine block at G1/S boundary.

Since HSET OE enhances cellular proliferation in HeLa cells, we were curious to examine any changes in the cell cycle kinetics of cells that stably overexpress HSET (HeLa-HSET-GFP cells) compared to the parental ones. To this end, we synchronized HeLa and HeLa-HSET-GFP cells using a single thymidine block (19h) followed by 2-color flow cytometric analysis of cell cycle profiles of HeLa-HSET-GFP and HeLa cells upon their release from the block at G1/S border. DNA content was analyzed with propidium iodide (PI) staining, where G2/M population was represented by double the intensity of PI (4N) compared to G1 cell population (2N). Anti-MPM-2 antibody tagged with Alexa488 secondary antibody was used to detect a mitosis-specific marker (MPM-2), in order to distinguish between 4N DNA-bearing G2 and M populations. A close interval cell cycle profiling revealed that HeLa-HSET-GFP cells demonstrated faster cell cycle progression kinetics; in other words, the duration of one complete cell cycle was reduced in HeLa-HSET-GFP cells (10.5h) as compared with wild-type cells (13h), with a stark shortening of the G2-and M-phases (Fig. 5A, B, C). This experiment was performed 3 times and the average time was represented as final duration of cell cycle phases in Fig. 5A. This trend was reflected when cyclin B1 levels (indicating mitotic phase) were followed in synchronized HeLa and HeLa-HSET-GFP cells using western blotting. While cyclin B1 levels surged at 10h followed by a decline in HeLa cells, they peaked at 8h and then declined in HeLa-HSET-GFP cells (Fig. 5D). Transient knockdown (KD) of HSET in HeLa cells resulted in a marginal increase in cell cycle duration (14h as compared to 13h in HeLa cells) with a protracted G2/M-phase (Suppl. Fig. 4A). This observation is in accordance with previously observed effects of HSET depletion in human fibroblast cells leading to delayed cyclin A degradation [28].

Most often, G1-phase contributes significantly to the cell cycle duration; thus, we sought to determine the effect of HSET OE and KD on G1-phase kinetics. Upon gradual decrease of serum concentration from 10% to 0% over 24h and an additional 12hr serum starvation, transiently transfected HeLa control vector (CV), HeLa HSET OE and HeLa HSET KD cells were replenished with serum-containing medium and stained with “*Cell-Clock*” dye (Biocolor; a redox dye that changes color corresponding to distinct phases in cell cycle). Yellow cells in the culture represent G1 and their color changes to light green in S-phase. We followed the proportion of G1 (yellow-colored) cells from 0h (50-70% G1 enrichment) to 9h after serum replenishment in all the three cases (CV, OE and KD). We observed negligible differences in the proportion of G1 cells in all three conditions (Suppl. Fig. 4Bi, Bii). This suggests that unlike G2-and M-phase kinetics, HSET OE does not significantly affect the duration of G1-phase.

**Figure 6:**
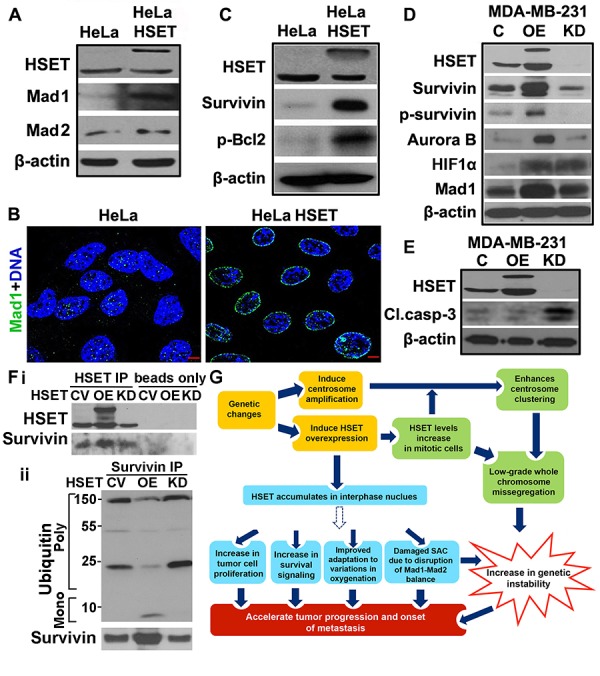
HSET overexpression upregulates survival proteins and disrupts balance of checkpoint proteins (A) Immunoblots showing HSET, Mad1 and Mad2 protein levels in HeLa and HeLa-HSET-GFP cells. β-actin is used as a loading control for all western blots. (B) Immunofluorescence micrographs showing Mad1 (green) levels and localization in HeLa and HeLa-HSET-GFP cells. (C) Immunoblots showing the expression levels of survival proteins (survivn, p-Bcl2) in HeLa and HeLa-HSET-GFP cells. (D) Immunoblots showing the expression of proteins associated with cell survival, cell cycle regulation, spindle assembly checkpoint and adaptation to hypoxia in MDA-MB-231 cells (C) compared to MDA-MB-231 cells transiently transfected with HSET-pEGFP plasmid (OE) or HSET siRNA (KD). (E) Immunoblots showing HSET and cleaved caspase-3 protein expression in MDA-MB-231 cells transiently transfected with vehicle control, HSET pEGFP plasmid or HSET siRNA, followed by UV-C exposure at 25 J/m^2^ for 10 min. (Fi) Immunoblots showing HSET and survivin protein levels in MDA-MB-231 with control vector (CV), with HSET overexpression and with HSET knockdown when HSET was immunoprecipitated (HSET IP) or not immunoprecipitated (beads only) followed by immunoblotting against survivin. (Fii) Immunoblots showing survivin immunoprecipitated from MDA-MB-231 cells (CV, OE and KD) and imuunoblotted agaist surviving and ubiquitin. (G) Schematic model depicting the involvement of HSET in tumor progression and metastasis via a) previously established mitotic pathways (Green boxes) and interphase-specific pathways suggested by our data (blue boxes). Dotted arrow indicates an unknown and indirect modulation of various downstream pathways by overexpressed nuclear HSET. C= control GFP vector.

Faster kinetic progression of HeLa-HSET-GFP cells (through G2- and M) compared to HeLa cells raised the possibility that G2/M or spindle assembly checkpoint (SAC) functions may be compromised in HeLa-HSET-GFP cells. Mad1 is a critical component of the SAC along with Mad2, and an imbalance in the Mad1-Mad2 protein ratio results in a damaged SAC causing premature anaphase entry and chromosome instability [29, 30]. Interestingly, we found that HeLa-HSET-GFP cells express markedly higher levels of Mad1 with a distinct nuclear envelope localization compared with parental HeLa cells (Fig. 6A, B). This observation along with the known association of HSET with importins, indicate that HSET might be involved in regulating mitotic entry/exit and nuclear export [31]. By contrast, there was no significant difference in the levels of Mad2 between the two cell lines (Fig. 6A), showing that the Mad1-Mad2 balance is highly perturbed in the HeLa-HSET-GFP cells. Thus we envision that excess HSET directly or indirectly incapacitates the SAC by disrupting the Mad1/Mad2 balance. The HeLa-HSET-GFP cells thus progress through the cell cycle rapidly in the presence of compromised checkpoints, which precipitates a greater likelihood of generating aneuploidy and, thus, may accelerate the process of tumor evolution.

Our data from the HeLa-HSET-GFP cells demonstrate that HSET OE can markedly accelerate the kinetics of G2 and M (Fig. 5A, B, C). Intriguingly, our immunohistochemical data from clinical tumor samples (Fig. 3A) showed strong nuclear localization of HSET. In order to obtain a deeper understanding of how elevated HSET levels may hasten progression through G2-and M-phases of the cell cycle and to exclude the possibility that faster kinetic progression through the cell cycle may result from artifactual mislocalization of HSET, we decided to examine in detail the sub-cellular localization of this intriguing protein in HeLa cells at various cell cycle stages. We found that HSET is conspicuously confined to the nucleus throughout interphase (Suppl. Fig. 5). Our observations are consistent with the finding that the *Xenopus* homolog of HSET, XCTK2, is sequestered in the nucleus in interphase in a Ran-dependent manner via the association of the NLS of XCTK2 with importin α/β [32]. In summary, the nuclear localization of the human HSET protein in interphase strongly suggests that the acceleration of the kinetics of G2 may be ascribed to a hitherto unknown activity of HSET within the nucleus.

### HSET overexpression upregulates survival signaling in cancer cells

Tumor cell numbers and tumor growth are not only a function of rate of cell proliferation but are also crucially influenced by cell survival and/or apoptosis. Having ascertained that HSET OE can enhance the kinetics of cell proliferation in tumors, we wanted to investigate whether elevated levels of HSET have any impact on the status of pro-survival signaling in HeLa cells. Immunoblots showed enhanced survival signaling as evidenced by notably high survivin and p-Bcl2 levels in HeLa-HSET-GFP cells (Fig. 6C) compared with levels seen in parental HeLa cells. To investigate if HSET OE affects signaling pathways that impinge on cell proliferation, or cell survival in breast cancer cells, we compared levels of some key proliferation, hypoxia and cell survival markers in parental MDA-MB-231 cells with MDA-MB-231-HSET overexpressing cells and MDA-MB-231-HSET knockdown cells. We observed enhanced levels of survivin and phospho-survivin, the hypoxia-induced factor HIF1α, the SAC protein Mad1 and the mitotic kinase Aurora-B in MDA-MB-231-HSET overexpressing cells (Fig. 6D). However, upon HSET knockdown, marginal or no reduction was observed in the expression levels of these proteins as compared with their respective levels in control cells (Fig. 6D). We also observed enhanced Aurora-B kinase activity as well as elevated expression of cyclin B1, D and A upon HSET overexpression in MDA-MB-231 cells (Suppl. Fig. 6A). The differential effects observed upon HSET OE and KD indicated that HSET may not normally be a key regulator of proliferation and survival pathways. Several studies [33] have in fact shown that HSET function is dispensable for the viability of most non-cancerous cells. However, our OE data strongly suggest that an elevated level of HSET expression thrusts proliferation and survival signaling in cancer cells into an “overdrive” mode. Using immunohistochemical analysis, we also confirmed that HSET nuclear WI correlated strongly with survivin WI (r=0.68, p=0.05) and Ki67 WI (r=0.32, p<0.001) in clinical samples (n=163). In summary, while HSET plays a non-essential role in regulating survival signaling in cancer cells, HSET overexpression enhances both proliferation as well as survival of cancer cells and perhaps fuels tumor progression by providing cancer cells with a proliferation and survival advantage. Our data thus provide evidence that cancer cells may employ auxiliary pathways/mechanisms, such as those involving the kinesin motor HSET, to their advantage.

To further explore the physiological role of HSET in cell survival signaling, we assessed the ability of MDA-MB-231 cells with HSET OE or KD to resist UV-induced apoptosis. To this end, we transiently transfected MDA-MB-231 cells with control vector, HSET OE construct or HSET siRNA (~70% transfection efficiency) 24h prior to UV irradiation. Following 10 min exposure to UV-C at 25 J/m^2^, cells were placed in the incubator for apoptosis induction for 5h. Lysates were then collected for determining HSET and cleaved caspase-3 protein levels (an early marker for apoptosis induction) and cell viability was determined using trypan blue assay. Western blot analysis revealed significantly higher cleaved caspase-3 induction in cells with HSET knock-down, whereas cells with HSET OE showed slightly lower cleaved caspase-3 levels as compared to cells transfected with control vector (Fig. 6E). These data indicate the ability of HSET overexpression to promote cell survival in cancer cells.

### HSET overexpression increases steady-state survivin levels by decreasing its poly-ubiquitination

Since we observed extensive upregulation of survivin protein expression as a result of HSET OE and significant reduction upon HSET KD, we wanted to determine if HSET occurs in the same protein complex as survivin and whether HSET OE has any effect on the APC/C-dependent proteolysis of survivin.

First, we tested if HSET and survivin co-immunoprecipitate with each other. We immunoprecipitated HSET from whole cell lysates of MDA-MB-231 cells that carried (i) a control vector, (ii) an HSET OE plasmid, and (iii) an HSET siRNA-bearing construct. Upon probing the immunoprecipitates for survivin, we confirmed that the anti-HSET antibody was able to pull down survivin in all the three cases, with an increased survivin pull down in the cell lysates overexpressing HSET (Fig. 6Fi). We also confirmed this association by immunoprecipitating survivin and in turn probing with HSET antibody (Suppl. Fig. 6B). These data indicate that HEST binds to survivin either directly or indirectly.

Since survivin's role in prosurvival signaling is strongly regulated by its degradation via ubiquitination [34], we further set out to test the possibility that increased HSET binding to survivin protects survivin from ubiquitination and APC/C-dependent degradation. In MDA-MB-231 cells transiently transfected with control vector, HSET-GFP plasmid and HSET siRNA, we immunoprecipitated survivin and immunoblotted against survivin and ubiquitin. Intriguingly, we observed reduced polyubiquitin signals in HSET overexpressing cells, even though survivin protein levels were extensively overexpressed in HSET overexpressing cells (Fig. 6Fii) as observed earlier (Fig. 6D). We also observed marginally higher ubiquitin levels in HSET KD cells as compared to control, even though the survivin protein levels were comparable in both the cases (Fig. 6Fii). These observations, in sum, uncover a previously unrecognized role of HSET overexpression in tumor progression via supplementing prosurvival pathways (Fig. 6G).

## DISCUSSION

In recent years, the key role played by the kinesin-14 protein HSET/KifC1 in centrosome clustering in cancer cells with supernumerary centrosomes, has been well established. In addition to its mitotic spindle-specific roles, several other roles of HSET requiring or independent of its motor activity have been suggested such as a role in processing early endocytic vesicles [35], rat spermatogenesis [36] and active transport of bare double-stranded DNA [37]. Although it is presently unclear whether HSET performs all these functions in cancer cells, these studies bring to light the possibility that HSET's involvement in tumor biology could be multifaceted.

We (Fig. 1A,B) and others [9] have found that a variety of primary tumors overexpress HSET as compared to their normal adjacent tissues. Several other threads of largely correlative and circumstantial evidence have suggested an involvement of HSET in driving tumor progression and metastases [9, 11]. However, our study is the first to explore and obtain several new mechanistic insights into the pathology of excess HSET in breast cancer cells. We have firmly cemented the hitherto anecdotal evidence with experimental data to show that HSET OE in breast cancer (i) correlates strongly with aggressiveness of the disease, (ii) is attributable, at least in part, to amplification of the genomic locus for this gene, and (iii) promotes tumor cell proliferation by accelerating cell cycle kinetics. Given the myriad of clinical implications of these important findings, our study spotlights the tremendous potential that HSET presents both as a biomarker of tumor progression and as an invaluable cancer cell-specific therapeutic target.

Four critical observations lead us to believe that HSET might have additional roles in driving tumor progression, independent of its centrosome clustering/spindle pole focusing role in mitosis, viz., (i) elevated ‘nuclear’ expression of HSET predominantly in the interphase cells within high grade tumors as revealed by immunohistochemical staining suggests that HSET may perform critical mitosis-independent functions in aggressive tumors or plausibly lead to more aggressive phenotypes within tumors; (ii) overexpression of HSET results in accelerated G2- and M-phases. Faster mitoses can conceivably arise from severely compromised SAC function that presumably allows HSET-overexpressing cells to rapidly traverse mitosis in the presence of aberrations including chromosome attachment errors. However, we are aware of the caveat that this mitotic role of HSET does not provide an alibi for the observed faster progression through the G2-phase upon HSET overexpression; (iii) HSET OE in HeLa cells leads to faster cell-cycle kinetics and enhanced overall proliferation (Fig. 5A, B, C), and (iv) HSET OE leads to the upregulation of the expression of phospho-survivin, Bcl-2, HIF1α, Aurora-B and Mad1, and presumably upregulates the signaling pathways that lie downstream of these key regulatory factors. Furthermore, since fewer than 3% of HeLa cells possess amplified centrosomes (our unpublished observations), we believe that the pro-proliferative role of HSET that we have demonstrated in our study in HeLa cells provides strong evidence for a centrosome clustering-independent activity of HSET.

To further support the centrosome clustering-independent aspect of HSET's role in driving tumor survival and proliferation, we assessed the effects of HSET OE and KD in HeLa cells with or without centrosome amplification. We induced extra centrosomes in HeLa cells by aphidicolin treatment (20 μM for 48h) and then compared the effect of HSET OE on expression of proliferation/survival markers in the HeLa cell lines bearing normonumerary and supernumerary centrosomes. The fact that we were able to show higher expression of proliferation and survival proteins upon HSET OE in the same cell line regardless of its centrosome status (Suppl. Fig. 7), asserts the centrosome clustering-independent role of HSET in driving cell proliferation and survival. Our data showed that HSET OE leads to an increase in Mad1 levels without any significant change in the levels of its partner protein, Mad2. Mad1 overexpression in HeLa cells has been shown to disrupt the stoichiometric balance between Mad1 and Mad2 to severely cripple SAC function leading to aneuploidy and chromosomal instability [29, 30]. We postulate that this surge in Mad1 protein levels (Fig. 6A,) facilitates premature anaphase entry by titrating the soluble pool of Mad2 and thereby damaging SAC function, and provides a possible explanation for the speedier execution of mitosis in HeLa-HSET-GFP cells. In addition, our study has yielded several novel mechanistic insights regarding the signaling pathways governed by HSET. Our data indicate that HSET is actually a key member of an oncoprotein axis that includes HIF1α and Aurora-B, and controls survival signaling through phospho-survivin and Bcl-2 (Fig. 6A, C, D). The dysregulation of HIF1α and Aurora-B are implicated in many aspects of cancer development and advancement [38, 39]. Notably, HIF1α drives the expression of survivin, which performs a dual function: it is an anti-apoptotic protein that additionally promotes cell proliferation [40-42]. Aurora-B is a chromosomal passenger protein involved in chromosome segregation, the spindle checkpoint and cytokinesis [43]. Aurora-B overexpression, observed in several tumor types [44], has been linked with aggressive metastasis and poor prognosis of cancer patients [45, 46]. Our data thus suggest that HSET OE-driven elevation in HIF1α and Aurora-B kinase levels incites upregulation of the pro-proliferative and pro-survival signaling networks and together with the increased aneuploidy triggered by impaired SAC function, facilitates tumor evolution into more malignant forms.

Importantly, our immunoprecipitation experiments demonstrate that both HSET and survivin exist within the same complex in MDA-MB-231 cells. We further investigated the molecular and functional significance of HSET's association with survivin and uncovered that HSET binding to survivin protects survivin from degradation by interfering with the latter's ubiquitination. It has been shown that survivin ubiquitination and degradation occurs in the nucleus. We propose that high levels of nuclear HSET inhibit the ubiquitination-dependent proteolysis of survivin in the interphase nucleus of cancer cells. Survivin accumulation is known to increases Aurora-B kinase activity, which in turn,vincrease the endogenous levels of phosphorylated histone H3; clearly, we observe all these effects following HSET OE (Fig. 6). Thus, we have provided mechanistic evidence that HSET OE, by stabilizing survivin, leads concurrently to both increased cell proliferation and survival signaling.

A recent study revealed HSET (along with other cell cycle regulated genes) as a transcriptional target of p110CUX1 [47]. Constitutive activation of p110CUX1 is known to drive cell proliferation by expediting entry into S-phase [48]. Interestingly, Mad1 is also shown to be transcriptionally regulated by p110CUX1 [49]. In light of these insights, we are unable to rule out the possibility that HSET nuclear overexpression and upregulation of Mad1 levels are a mere consequence of an upstream regulation by classical tumor promoting genes. The Cux1-E2F-HSET cell proliferation axis thus demands further exploration in order to substantiate the validity of this prospect. Besides, we cannot discount the significance of substantial cell cycle effects observed upon HSET overexpression and the unyielding relationship between HSET nuclear expression and patient survival.

Taken together, our results provide compelling evidence that HSET OE drives tumor progression through multiple mechanisms that include (i) enhancement of tumor cell proliferation rates, (ii) increasing aneuploidy through centrosome clustering, upregulation of Aurora-B and compromised SAC function and (iii) promoting pro-survival signaling. Clearly, these findings that argue for the existence of a causative link between nuclear HSET accruement and tumor aggressiveness, have far-reaching clinical implications, including unlocking the potential of HSET nuclear expression serving as a prognostic biomarker, and HSET taking shape as a cancer-selective therapeutic target for the design and preclinical development of small-molecule HSET inhibitors for non-toxic breast cancer therapy.

## MATERIALS AND METHODS

### *In silico* analysis of HSET gene expression

One channel micro array data were collected from the Gene Expression Omnibus (GEO) database and processed using Robust Multiarray (RMA) normalization and were further used for gene expression analysis. The list of the GSE ID's are given in Supp. Table 1. Log2-transformed HSET expression levels are plotted in Fig. 1 for each of the glioblastoma, lung, breast, colon cervical cancer and leukemia patients compared with their normal pairs. Statistical analysis was performed using Student's t-test. The criterion for statistical significance was p< 0.05.

### Clinical tissue samples

All paraffin-embedded tissue slides were commercially obtained (from Accumax and US Biomax). A subset of well-annotated tissue microarrays (339 biospecimens) with information on clinical outcomes (n=163) were obtained from Emory University Hospital. The Emory Institutional Review Board approval was obtained for all aspects of the study.

### Immunohistochemistry, scoring and statistics

For immunohistochemical staining, the TMAs were first deparaffinized and then rehydrated in a series of ethanol baths (100%, 90%, 75% and 50%). Antigen retrieval was achieved by citrate buffer (pH 6.0) in a pressure-cooker (15 psi) for 30 min. Immunostaining for HSET (1:1000 dilution) was performed using a rabbit polyclonal antibody which was a generous gift from Claire Walczak (Indiana University). Enzymatic antibody detection was performed using Universal LSAB + kit/HRP (DAKO, CA, USA). HSET staining was scored for both the nuclear and cytoplasmic localization as an intensity and frequency score by an experienced pathologist. A relative intensity score was represented as 0 = none, 1 = low, 2 = moderate, or 3 = high and frequency score was represented as the percentage of cell nuclei or cytoplasms demonstrating HSET positivity.

### Statistical methods

Statistical analyses were performed using SAS Version 9.3 with HSET WI considered as a continuous variable in all the required analysis tests. Kaplan-Meier survival curves were generated for patient outcomes (OS and PFS) stratified by negative and positive HSET WI groups. Survival differences between the groups were assessed using the log-rank test.

### Cell culture and transfection

HeLa-HSET-GFP cells were generously provided by Claire Walczak (Indiana University). HeLa, HeLa-HSET-GFP and MDA-MB-231 cells were grown in DMEM supplemented with 10% FBS and 1% penicillin/streptomycin. Briefly, cells were seeded onto 100-mm plates 1 day prior to transfection. Plasmid DNA (5 μg) and 15 μl of DharmaFECT 4 transfection reagent (Thermo Scientific, PA, USA) were used for each transfection. HSET-pEGFP plasmid was generously provided by Dr. Walczak. Cells overexpressing HSET were selected in the medium containing G418 (400 μg/ml). The G418-resistant colonies were collected and examined for HSET expression. SMARTpool: ON-TARGETplus KIFC1 siRNA (Dharmacon, PA, USA) was used to knockdown HSET in MDA-MB-231 cells.

### Cellular protein preparation, western blotting, immunofluorescence and antibodies

Cells were cultured to ~70% confluence and protein lysates were collected following transfection or otherwise. Fresh frozen tissue sections were first sonicated and lysates were then prepared. The immune-reactive bands corresponding to the respective primary antibodies were visualized by the Pierce ECL chemiluminescence detection kit (Thermo Scientific). β-actin was used as loading control. For immunofluorescence staining, cells grown on glass coverslips were fixed with −20 °C methanol for 10 min and blocked by incubating with 2% bovine serum albumin/PBS/0.05% Triton X-100 at 37 °C for 1h. Specific primary antibodies were incubated with coverslips for 1h at 37 °C at the recommended dilution. The cells were washed with 2% bovine serum albumin/PBS for 10 min at room temperature before incubating with a 1:2000 dilution of Alexa 488- or 555-conjugated secondary antibodies. Cells were mounted with Prolong Gold antifade reagent that contains 4′,6-diamidino-2-phenylindole (Invitrogen). Polyclonal rabbit anti-HSET antibody was provided by Dr. Claire Walczak. Antibodies against α-tubulin and β-actin were from Sigma (St. Louis, MO, USA). Antibodies against γ-tubulin, α-tubulin and β-actin were from Sigma (St. Louis, MO, USA). Anti-Mad2 antibody was from BD Biosciences (Pharmingen, San Jose, CA, USA). Antibodies against p-Bcl2 and cleaved caspase-3 were from Cell Signaling (Danvers, MA, USA). Alexa 488- or 555- conjugated secondary antibodies were from Invitrogen (Carlsbad, CA, USA). Anti-Mad1 antibody was a generous gift from Andrea Musacchio affiliation. Anti-Ki67 antibody was from Abcam (Cambridge, MA, USA). Horseradish peroxidase-conjugated secondary antibodies were from Santa Cruz Biotechnology (Santa Cruz, CA, USA).

### Kinase activity assay

To examine cdk1 and Aurora-B kinase activity, respective primary antibodies were used to selectively immunoprecipitate protein-containing complexes from cell lysates. The resulting immunoprecipitate was incubated with pure histone-H3 protein in the presence of ^32^P-labelled ATP and kinase buffer. The kinase assay reaction allowed immunoprecipitated cdk1 to phosphorylate histone-H3 *in vitro*, the extent of which was measured by immunoblotting using phosphohistone-H3 antibody from Cell Signaling (MA, USA). Histone-H3 protein was from Millipore (MA, USA) and ATP was from Cell Signaling.

### Fluorescence *in situ* hybridization

The samples from tumor cell lines or tumor tissue were hybridized by 2-color FISH with an HSET-specific BAC probe (RPCI-11 602P21, green) and a chromosome 6 centromere probe (CH514-7B4, red) (BACPAC). The HSET and centromere 6 probes were labeled with Cy3-dUTP (red) and FITC-dUTP (green), respectively, and hybridized with nuclei from cell lines or tumor tissue samples. Plasmids for production of a particular FISH probe were combined in equimolar amounts (55–70 pM). Nick translation was performed on 2 μg of this substrate by using Nick translation kit (Abbott Molecular, IL, USA). The translation product was denatured for 3 mins at 95°C followed by fast cooling on ice and confirmed in 1.5% agarose gel electrophoresis as a smear of fragments ranging between 100 and 300 bp. A 2 min denaturation at 76°C was followed by overnight (12–16h) incubation at 37°C. Hybridization of the FISH probes was carried out in LSI/WCP hybridization buffer (Abbott Molecular, IL, USA). The slides were counterstained with DAPI (Invitrogen, NY, USA) and Zeiss LSM 700 confocal microscope was used to capture FISH images. Results were expressed as a ratio of the number of copies of the HSET gene to the number of chromosome 6–centromeric markers.

### Flow cytometry

Trypsinized cells were resuspended in PBS at 10^6^ cells/ml. Cells were then fixed by addition of ice-cold 70% ethanol. Ethanol-fixed cells were kept overnight at 4°C before staining. Cells were pelleted and washed twice with PBS. Cell pellets were incubated for an hour at room temperature with mouse anti-MPM-2 antibody (Millipore, MA, USA), followed by1h incubation with Alexa-488 anti-mouse secondary antibody (Life Technologies, NY, USA). Finally cells were washed, pelleted and resuspended in PI containing isotonic buffer (0.1 mg/ml) and 0.5% Triton X-100. Cell cycle distribution was determined by flow cytometry using an LSRFortessa Flow cytometer (BD Biosciences, CA, USA) and analyzed using Flowjo software (Tree Star, OR, USA).

### Trypan Blue cell exclusion assay

Cells were cultured to ~70% confluence followed by centrifuging and pellet was resuspended in 1 ml culture medium. 0.1 mL of 0.4% trypan blue solution was then added to 1 mL of cell suspension. A hemacytometer was loaded with 10 μl of the solution and examined immediately under a microscope. Live (white) and dead (blue) cells were counted and percent cell viability was calculated using the following formula: percent viable cells = [1.00 − (Number of live cells ÷ Number of total cells)] × 100.

### BrdU incorporation assay

Asynchronous proliferating HeLa and HeLa-HSET-GFP cells were grown on coverslips to a confluency of ~70% and then incorporated with 10μM BrdU for 1h followed by fixation with 70% ethanol at room temperature and immersion in 0.07 N NaOH for 2 minutes (which was then neutralized with PBS, pH 8.5). Coverslips were then incubated in 2% bovine serum albumin/PBS.0.05% Triton X-100 at 37 °C for 1h followed by immunostaining using a 1:1000 dilution of Anti-BrdU-FITC antibody (BD Biosciences, San Jose, CA, USA). BrdU positive cells, indicative of cell proliferation, were captured on a Zeiss Axioplan-2 fluorescence microscope (20X objective).

### Immunoprecipitation and endogenous ubiquitination analysis

MDA-MB-231 cells were transiently transfected with control vector, HSET-pEGFP plasmid or SMARTpool siRNA as described above and lysates were collected. Cell lysates were clarified by centrifugation at 10,000 rpm, and the supernatants (500 μg of protein) were subjected to immunoprecipitation with 4 μl of anti-HSET or anti-survivin antibodies. After overnight incubation at 4°C, protein A-agarose beads were added and left at 4°C overnight. Immunocomplexes were then subjected to Western blot analysis as described previously. Western blot analysis with anti-ubiquitin antibody (Life Sensors, PA, 1:500) was performed by first incubating the PVDF membrane with 0.5% glutaraldehyde/PBS pH 7.0 for 20 min and then probing for the antibody.

## SUPPLEMENTARY MATERIAL FIGURES AND TABLE


