# Supplementary material for: HSET overexpression fuels tumor progression via centrosome clustering-independent mechanisms in breast cancer patients
Source: Oncotarget. 2015 Feb 28;6(8):6076–91. doi: 10.18632/oncotarget.3475 (PMC4467423; doi:10.18632/oncotarget.3475)
Supplement: Supplementary file 1 [file oncotarget-06-6076-s001.pdf]

HSET overexpression fuels tumor progression via centrosome clustering-independent mechanisms in breast cancer patients

Supplementary Material

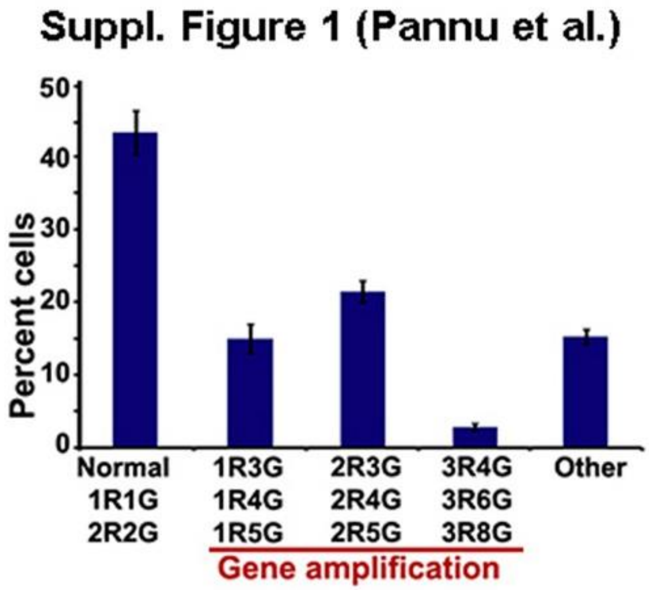

Suppl. Fig. 1: HSET gene amplification in fresh human breast tumor tissue. Bar graph representation of various combinations of red and green copy numbers observed for HSET locus and chromosome 6 centromere as determined by visual quantitation from confocal images in primary cells isolated from fresh tumor tissue ($p<0.05$).

Suppl. Figure 2 (Pannu et al.)

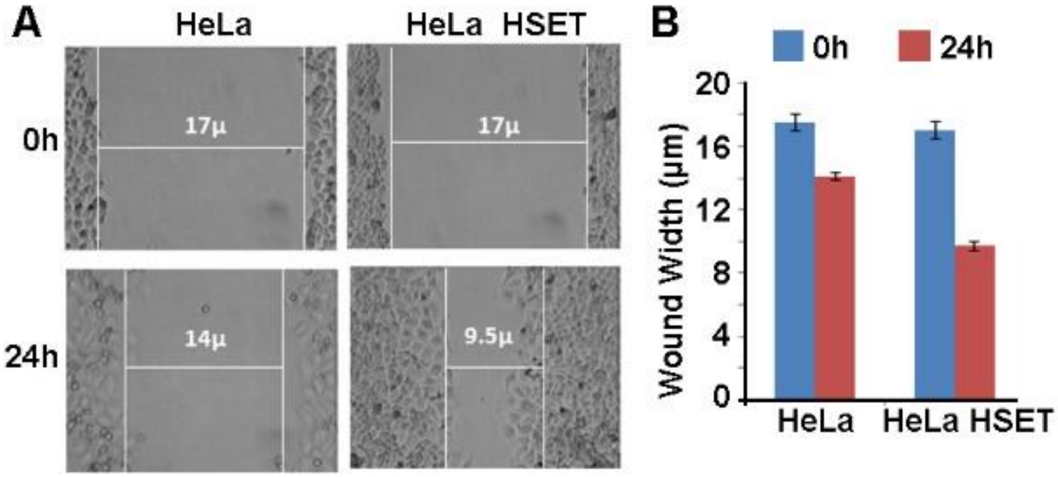

Suppl. Fig. 2: Cells overexpressing HSET show higher migration. A. Brightfield microscopic images showing wound healing capacity of HeLa cells overexpressing HSET or wild-type HeLa cells at 0h and 24h. B. Bar graph showing the wound width at 0h and 24h after making the scratch. $p<0.05$.

Suppl. Figure 3 (Pannu et al.)

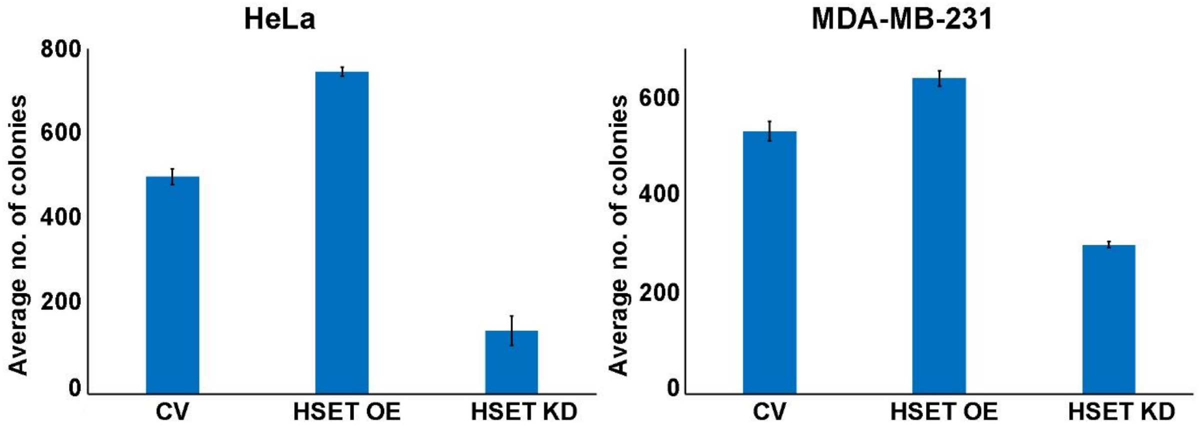

Suppl. Fig. 3: Colony formation assay in HeLa and MDA-MB-231 cells with HSET OE and KD. Bar graphs representing average number of colonies counted 72 h after the transfected cells were seeded (2000 cells per well). Cells were stained with crystal violet, colonies were counted manually and average of 3 wells was plotted as bar graphs. $p<.005$

Suppl. Figure 4 (Pannu et al.)

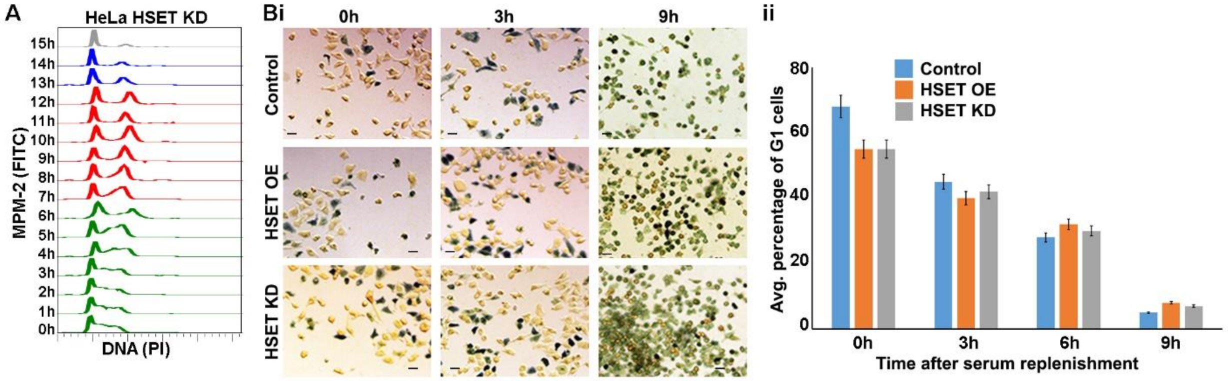

Suppl. Fig. 4: Cell cycle kinetics in HeLa cells upon HSET OE and KD. (A) Cell cycle histograms representing cell cycle profiles of synchronized HeLa-HSET-KD cells from the point of thymidine block release (0h) to the point after mitotic exit (15h). Green lines represent S phase, red lines represent G2 phase and blue lines represent M phase. (Bi) Micrographs showing HeLa cells (CV, OE and KD) in different phases of cell cycle when released from serum starvation by using Cell-Clock assay kit. Yellow color depicts G1 phase cells, yellowish-green color depicts S phase, light blue color depicts G2 phase and dark blue color depicts M phase. (Bii) Bar graphs representing average percentage of cells in G1 phase out of total cells counted in 5 random fields, from 0h to 9h after serum replenishment. $p<.005$.

Suppl. Figure 5 (Pannu et al.)

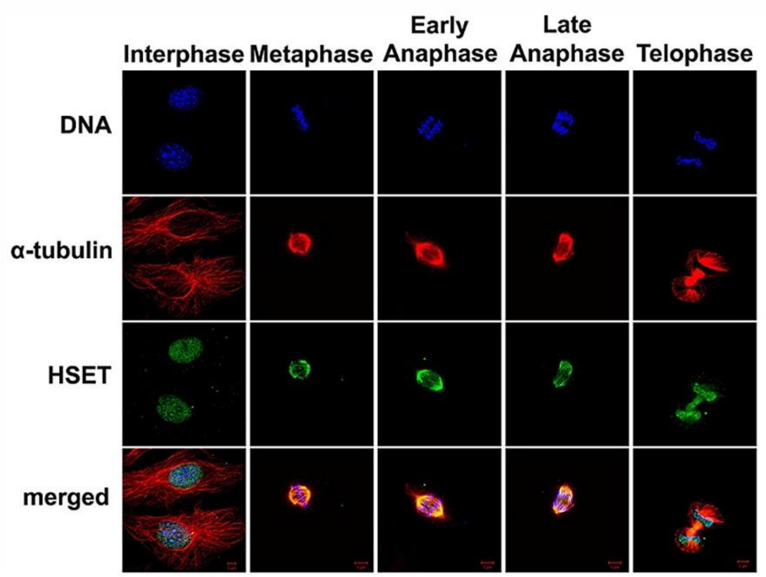

Suppl. Fig. 5: Confocal micrographs showing HSET localization in various phases of cell cycle. HeLa cells were co-immunostained with HSET (green) and α-tubulin (red) antibodies. DNA was stained with DAPI (blue). Nuclear localization of HSET is clearly visible in interphase and telophase, whereas it is seen to be localized on minus-ends of microtubules in mitotic spindles during metaphase and anaphase. Scale bar 5μm.

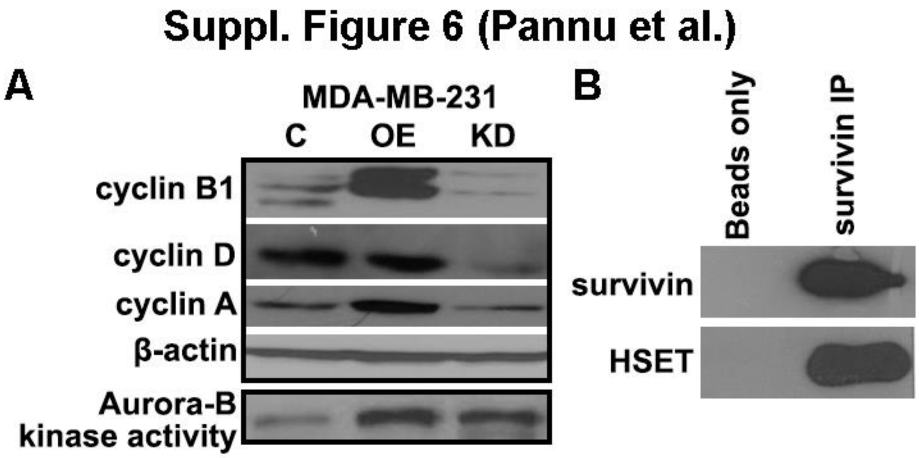

Suppl. Fig. 6: A. Immunoblots showing the expression of proteins associated with cell cycle regulation in MDA-MB-231 cells (C) compared to MDA-MB-231 cells transiently transfected with HSET-pEGFP plasmid (OE) or HSET siRNA (KD). B. Immunoblots showing HSET and survivin protein levels in MDA-MB-231 when surviving was immunoprecipitated (survivin IP) or not immunoprecipitated (beads only) followed by immunoblotting against HSET.

Suppl. Figure 7 (Pannu et al.)

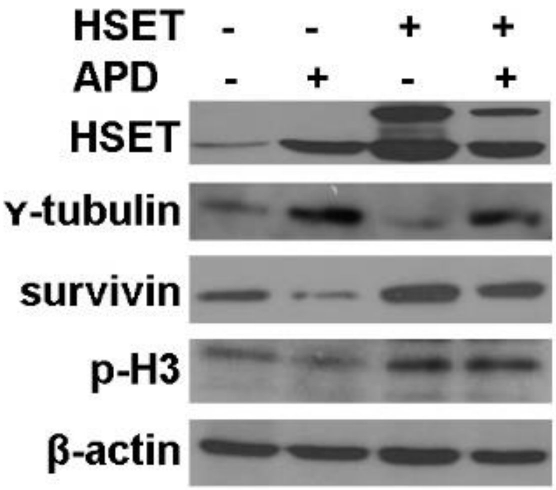

Suppl. Fig. 7: Proliferation and survival effects of HSET OE in HeLa cells with or without amplified centrosomes. Immunoblots showing centrosome amplification (indicated by accumulation of centrosomal γ-tubulin), upregulated survival signaling (indicated by increased survivin levels) and proliferation (increase in p-H3 levels) markers in HeLa cells under indicated conditions. β-actin was used as loading control. APD: aphidicolin.

Suppl. Table 1: List of the GSE ID's for GEO database used in analysis.

**Cancer Type**	**Normal Samples GEO Series ID**	**Cancer Samples GEO Series ID**	**N Normal**	**N Cancer**
Glioblastoma	GSE10878	GSE10878	3	20
Lung Cancer	http://www.broadinstitute.org/mpr/publications/projects/Lung Cancer/		17	19
Leukemia	http://www.broadinstitute.org/mpr/publications/projects/Leukemia/		16	6
Breast Cancer	GSE10797	GSE7390, GSE18864	16	179
Colon Cancer	GSE4107	GSE18088	10	53
Cervical Cancer	GSE9750	GSE9750	21	33
